# Clinico‐radiological features, molecular spectrum, and identification of prognostic factors in developmental and epileptic encephalopathy due to inosine triphosphate pyrophosphatase (ITPase) deficiency

**DOI:** 10.1002/humu.24326

**Published:** 2022-01-12

**Authors:** Marcello Scala, Saskia B. Wortmann, Namik Kaya, Menno D. Stellingwerff, Angela Pistorio, Emma Glamuzina, Clara D. van Karnebeek, Cristina Skrypnyk, Katarzyna Iwanicka‐Pronicka, Dorota Piekutowska‐Abramczuk, Elżbieta Ciara, Frederic Tort, Beth Sheidley, Annapurna Poduri, Parul Jayakar, Anuj Jayakar, Jariya Upadia, Nicolette Walano, Tobias B. Haack, Holger Prokisch, Hesham Aldhalaan, Ehsan G. Karimiani, Yilmaz Yildiz, Ahmet C. Ceylan, Teresa Santiago‐Sim, Amy Dameron, Hui Yang, Mehran B. Toosi, Farah Ashrafzadeh, Javad Akhondian, Shima Imannezhad, Hanieh S. Mirzadeh, Shazia Maqbool, Aisha Farid, Mohamed A. Al‐Muhaizea, Meznah O. Alshwameen, Lama Aldowsari, Maysoon Alsagob, Ashwaq Alyousef, Rawan AlMass, Aljouhra AlHargan, Ali H. Alwadei, Maha M. AlRasheed, Dilek Colak, Hanan Alqudairy, Sameena Khan, Matthew A. Lines, M. Ángeles García Cazorla, Antonia Ribes, Eva Morava, Farah Bibi, Shahzad Haider, Matteo P. Ferla, Jenny C. Taylor, Hessa S. Alsaif, Abdulwahab Firdous, Mais Hashem, Chingiz Shashkin, Kairgali Koneev, Rauan Kaiyrzhanov, Stephanie Efthymiou, Queen Square Genomics, Thomas Schmitt‐Mechelke, Andreas Ziegler, Mahmoud Y. Issa, Hasnaa M. Elbendary, Pasquale Striano, Fowzan S. Alkuraya, Maha S. Zaki, Joseph G. Gleeson, Tahsin Stefan Barakat, Jorgen Bierau, Marjo S. van der Knaap, Reza Maroofian, Henry Houlden

**Affiliations:** ^1^ Department of Neurosciences, Rehabilitation, Ophthalmology, Genetics, Maternal and Child Health Università Degli Studi di Genova Genoa Italy; ^2^ Pediatric Neurology and Muscular Diseases Unit IRCCS Istituto Giannina Gaslini Genoa Italy; ^3^ UCL Queen Square Institute of Neurology University College London London UK; ^4^ Amalia Children's Hospital Radboud University Nijmegen Nijmegen The Netherlands; ^5^ University Children's Hospital Paracelsus Medical University Salzburg Austria; ^6^ Department of Genetics King Faisal Specialist Hospital and Research Centre Riyadh Saudi Arabia; ^7^ Department of Translational Genomics, Center for Genomics Medicine King Faisal Specialist Hospital and Research Centre Riyadh Saudi Arabia; ^8^ Department of Child Neurology, Emma Children's Hospital, Amsterdam Leukodystrophy Center, Amsterdam University Medical Centers Vrije Universiteit and Amsterdam Neuroscience Amsterdam The Netherlands; ^9^ Clinical Epidemiology and Biostatistics Unit IRCCS Istituto Giannina Gaslini Genoa Italy; ^10^ Adult and Paediatric National Metabolic Service Starship Children's Hospital Auckland New Zealand; ^11^ Departments of Pediatrics and Clinical Genetics Academic Medical Centre Amsterdam The Netherlands; ^12^ Department of Molecular Medicine, Al‐Jawhara Centre for Molecular Medicine Arabian Gulf University Manama Kingdom of Bahrain; ^13^ Department of Medical Genetics The Children's Memorial Health Institute Warsaw Poland; ^14^ Department of Audiology and Phoniatrics The Children's Memorial Health Institute Warsaw Poland; ^15^ Secció d'Errors Congènits del Metabolisme‐IBC, Servei de Bioquímica iGenètica Molecular Hospital Clínic, IDIBAPS, CIBERER Barcelona Spain; ^16^ Department of Neurology F.M. Kirby Neurobiology Center, Boston Children's Hospital Boston Massachusettes USA; ^17^ Division of Epilepsy and Clinical Neurophysiology and Epilepsy Genetics Program Boston Children's Hospital Boston Massachusettes USA; ^18^ Department of Neurology Harvard Medical School Boston Massachusettes USA; ^19^ Nicklaus Children's Hospital Miami Florida USA; ^20^ Tulane University School of Medicine New Orleans Louisiana USA; ^21^ Institute of Medical Genetics and Applied Genomics University of Tübingen Tübingen Germany; ^22^ Institute of Human Genetics Technische Universität München Munich Germany; ^23^ Institute of Human Genetics Helmholtz Zentrum München Neuherberg Germany; ^24^ Department of Neurosciences King Faisal Specialist Hospital and Research Centre Riyadh Saudi Arabia; ^25^ Department of Medical Genetics Next Generation Genetic Polyclinic Mashhad Iran; ^26^ Molecular and Clinical Sciences Institute St. George's University of London, Cranmer Terrace London UK; ^27^ Innovative Medical Research Center Islamic Azad University, Mashhad Branch Mashhad Iran; ^28^ Pediatric Metabolic Diseases Clinic Dr. Sami Ulus Training and Research Hospital for Maternity and Children Ankara Turkey; ^29^ Department of Medical Genetics Ankara City Hospital Ankara Turkey; ^30^ GeneDx Gaithersburg Maryland USA; ^31^ Pediatric Neurology Department, Ghaem Hospital Mashhad University of Medical Sciences Mashhad Iran; ^32^ Department of Pediatrics Mashhad University of Medical Sciences Mashhad Iran; ^33^ Department of Pediatric Diseases Mashhad University of Medical Sciences Mashhad Iran; ^34^ Development and Behavioral Pediatrics Department Institute of Child Health and The Children Hospital Lahore Pakistan; ^35^ Neurosciences Department King Fahad Medical City Riyadh Saudi Arabia; ^36^ Department of Clinical Pharmacy King Saud University Riyadh Saudi Arabia; ^37^ Department of Biostatistics, Epidemiology and Scientific Computing KFSHRC Riyadh Kingdom of Saudi Arabia; ^38^ Medical Genetics, Department of Pediatrics Alberta Children's Hospital Calgary Canada; ^39^ Inborn Errors of Metabolism Unit Hospital Sant Joan de Déu Barcelona Spain; ^40^ Department of Clinical Genomics, Laboratory of Medicine and Pathology Center for Individualized Medicine, Mayo Clinic Rochester Minnesota USA; ^41^ Institute of Biochemistry and Biotechnology Pir Mehar Ali Shah Arid Agriculture University Rawalpindi Pakistan; ^42^ Izzat Ali Shah Hospital Lalarukh Wah Cantt Rawalpindi Pakistan; ^43^ NIHR Oxford BRC Genomic Medicine, Wellcome Centre for Human Genetics University of Oxford Oxford UK; ^44^ International University of Postgraduate Education Almaty Kazakhstan; ^45^ Department of Neurology and Neurosurgery Asfendiyarov Kazakh National Medical University Almaty Kazakhstan; ^46^ Department of Neuropaediatrics Children's Hospital, Cantonal Hospital Lucerne Switzerland; ^47^ Zentrum für Kinder und Jugendmedizin Heidelberg, Sektion Neuropädiatrie und Stoffwechselmedizin Universitätsklinikum Heidelberg Heidelberg Germany; ^48^ Clinical Genetics Department, Human Genetics and Genome Research Division National Research Centre Cairo Egypt; ^49^ Department of Anatomy and Cell Biology Alfaisal University Riyadh Saudi Arabia; ^50^ Department of Neuroscience, Rady Children's Institute for Genomic Medicine, Howard Hughes Medical Institute University of California San Diego California USA; ^51^ Department of Clinical Genetics, Erasmus MC University Medical Center Rotterdam The Netherlands; ^52^ Laboratory of Biochemical Genetics, Department of Clinical Genetics Maastricht University Hospital Maastricht The Netherlands; ^53^ Department of Functional Genomics, Center for Neurogenomics and Cognitive Research VU University Amsterdam The Netherlands

**Keywords:** congenital microcephaly, developmental and epileptic encephalopathy 35, heart disease, *ITPA*, ITPase, white matter abnormalities

## Abstract

Developmental and epileptic encephalopathy 35 (DEE 35) is a severe neurological condition caused by biallelic variants in *ITPA*, encoding inosine triphosphate pyrophosphatase, an essential enzyme in purine metabolism. We delineate the genotypic and phenotypic spectrum of DEE 35, analyzing possible predictors for adverse clinical outcomes. We investigated a cohort of 28 new patients and reviewed previously described cases, providing a comprehensive characterization of 40 subjects. Exome sequencing was performed to identify underlying *ITPA* pathogenic variants. Brain MRI (magnetic resonance imaging) scans were systematically analyzed to delineate the neuroradiological spectrum. Survival curves according to the Kaplan–Meier method and log‐rank test were used to investigate outcome predictors in different subgroups of patients. We identified 18 distinct *ITPA* pathogenic variants, including 14 novel variants, and two deletions. All subjects showed profound developmental delay, microcephaly, and refractory epilepsy followed by neurodevelopmental regression. Brain MRI revision revealed a recurrent pattern of delayed myelination and restricted diffusion of early myelinating structures. Congenital microcephaly and cardiac involvement were statistically significant novel clinical predictors of adverse outcomes. We refined the molecular, clinical, and neuroradiological characterization of ITPase deficiency, and identified new clinical predictors which may have a potentially important impact on diagnosis, counseling, and follow‐up of affected individuals.

## Graphical abstract

Phenotype‐genotype spectrum of epileptic encephalopathy due to ITPase deficiency.

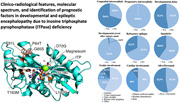

## 1 INTRODUCTION

Developmental and epileptic encephalopathy 35 (DEE 35; MIM# 616647) is a rare neurodegenerative condition characterized by developmental delay (DD), microcephaly, feeding difficulties, early‐onset refractory seizures (often within the first 6 months of life) followed by psychomotor stagnation/regression, and lethality in early childhood (Handley et al., 2019; Kaur et al., 2019; Kevelam et al., 2015). Cardiac and ocular involvement is frequently observed. White matter involvement is typical and consists of peculiar region‐specific abnormalities, predominantly involving early myelinating structures and suggestive of a neuronal degenerative process (Kevelam et al., 2015).

Biallelic variants in inosine triphosphate pyrophosphohydrolase (*ITPA*; MIM# 147520) have been first associated with DEE 35 in seven patients from four unrelated families by Kevelam et al. (2015). More recently, a few additional affected individuals have been reported (Bierau et al., 2007; Burgess et al., 2019; Burgis, 2016; Handley et al., 2019; Kaur et al., 2019; Rochtus et al., 2020; Sakamoto et al., 2020). Inosine triphosphate pyrophosphatase (ITPase), is an essential enzyme that removes the spontaneously arising noncanonical nucleotides inosine triphosphate (ITP) and deoxy‐inosine triphosphate (dITP) from the cellular nucleotide pool, playing a pivotal role in purine metabolism and cell function (Galperin et al., 2006; Holmes et al., 1979).

We report 28 unpublished individuals with DEE 35 from 23 unrelated families of different ancestry and review 12 previously reported cases. This clinical and neuroradiological characterization of a large cohort of 40 individuals allows a refined phenotypic description of ITPase deficiency. We further systematically investigate possible clinical predictors for adverse outcomes in this rare condition.

## 2 MATERIALS AND METHODS

### 2.1 Editorial policies and ethical considerations

This study adheres to the principles set out in the Declaration of Helsinki and was locally approved by the local Ethics Committees of the involved Institutions: Mayo Clinic, Rochester, 16‐004682; King Faisal Specialist Hospital and Research Centre, (2121053, 2120022, and 2161245); Technische Universitaet Muenchen, 5360/12 S; University College London, project ID: 07/N018, REC Ref: 07/Q0512/26). No IRB approval was necessary for retrospective data analysis of a single patient for the following Institutions: Alberta Children's Hospital, Calgary, Canada; Al‐Jawhara Centre for Molecular Medicine, Kingdom of Bahrain; Center for Neurogenomics and Cognitive Research, VU University, The Netherlands; Children's Hospital, Cantonal Hospital Lucerne, Switzerland; Dr. Sami Ulus Training and Research Hospital for Maternity and Children; Emma Children's Hospital, Amsterdam Leukodystrophy Center, The Netherlands; The Children's Memorial Health Institute, Poland. The authors obtained and archived written informed consents from parents or legal guardians of the enrolled subjects to publish genetic and clinical data, including brain magnetic resonance imaging (MRI) images (P1 and P3).

### 2.2 Patient enrolment

We ascertained the genotype and phenotype information for 28 novel subjects with severe epileptic encephalopathy. Patients were recruited through international collaboration, also using Genematcher (Sobreira et al., 2015), from several clinical and research centers in Europe, Africa, Middle East, North America, and New Zealand (for details see the Supporting Information Material). Written informed consent was obtained from the parents or legal guardians of all enrolled subjects. Phenotypes of two of these individuals (P9 and P14), who were partially described previously, have been extensively reported and updated (Bierau et al., 2007; Muthusamy et al., 2021).

### 2.3 Previously reported cases assessment

All articles indexed in PubMed (https://pubmed.ncbi.nlm.nih.gov/?term=itpa) between October 2015, when *ITPA* variants were first associated with DEE 35 by Kevelam et al. (2015), and March 2021 were retrieved using the terms “ITPA,” “ITPase deficiency,” and “epileptic encephalopathy 35.” All the articles were thoroughly reviewed concerning the molecular, clinical, and neuroradiological spectrum associated with DEE 35. Inclusion criteria for previously published patients were: availability of clinical data (with a focus on epilepsy, developmental, neuro‐, cardio‐ and ophthalmological findings), identification of (likely) pathogenic *ITPA* variants, lack of duplication from other previous reports. Exclusion criteria were: ambiguous clinical presentation not consistent with DEE 35 and inconclusive genetic testing.

### 2.4 Variant identification and analysis

Next‐generation sequencing panel for epileptic encephalopathies (P5 and P9) or exome sequencing (P1‐4, P6‐8, P10‐28) was performed on genomic DNA extracted from peripheral blood leukocytes (P1–15 and P17–28) or ORAcollect buccal swab (OCR‐100; DNA Genotek) (P16) using standard local protocols (Supporting Information Material). Chromosomal microarray analysis was performed in P2, P3, P26, and P27 according to standard methods (Shaw‐Smith et al., 2004). The identified variants were filtered according to minor allele frequency ≤0.001 in genomic databases (Genome Aggregation Database—gnomAD, Lek et al., 2016); Iranome, in‐house database of 16,000 control exomes, the Munich in‐house database (https://github.com/mri‐ihg/EVAdb), Great Middle Eastern Variome Project—GME), conservation (Genomic Evolutionary Rate Profiling—GERP, http://mendel.stanford.edu/SidowLab/downloads/gerp/), and predicted effect on protein structure and function. In silico prediction tools were used for the interpretation of candidate variants, including Combined Annotation Dependent Depletion (CADD; https://cadd.gs.washington.edu), Mutation Taster (http://www.mutationtaster.org), Sorting Intolerant From Tolerant (SIFT; https://sift.bii.a‐star.edu.sg), and Polyphen‐2 (http://genetics.bwh.harvard.edu/pph2/). Candidate variants were eventually classified according to the American College of Medical Genetics and Genomics and the Association for Molecular Pathology (ACMG/AMP) guidelines (Richards et al., 2015). Sanger sequencing was performed for validation and segregation analysis. All *ITPA* variants are reported according to RefSeq NM_033453.3, GenBank NC_000020.11. The change in protein stability was calculated with PyRosetta for the variants presented herein and the presumed neutral variants present in gnomAD (https://gnomad.broadinstitute.org/) (Chaudhury et al., 2010; Karczewski et al., 2020). All novel variants reported were deposited in the Leiden Open Variation Database (LOVD, https://www.lovd.nl) with the following accession numbers: #0000831814, #0000831817, #0000831819, #0000831820, #0000831821, #0000831822, #0000831823, #0000831824, #0000831825, #0000831826, #0000831827, #0000831828, #0000831829, and #0000831830. Further details are available in Supporting Information Material.

### 2.5 Neuroimaging analysis

Brain MRI scans were locally performed during routine patient care. Of these, 28 scans of 19 individuals were collected and analyzed in detail at the Amsterdam Leukodystrophy Center (The Netherlands). MRI scans of adequate quality, at least comprising T1‐weighted and transverse T2‐weighted images were systematically scored according to a previously published protocol by two independent authors (MDS and MSvdK) (van der Knaap et al., 1999). Additional sequences, such as diffusion‐weighted imaging (DWI), magnetic resonance spectroscopy, and contrast‐enhanced images were also evaluated, when available. MRIs were divided into four age groups (≤2, 2 to ≤4, 4 to ≤8, and >8 months) and neuroimaging features were systematically analyzed in each group of patients.

### 2.6 Statistical analysis

Descriptive statistics were performed first. Categorical variables were reported as absolute frequencies and percentages, and quantitative variables as median values and first and third quartiles (1st and 3rd q). For comparison of frequencies (e.g., frequency of deaths among males vs. females), the *χ* ^2^ test or Fisher's Exact test (in case of expected frequencies <5) was used. Survival curves according to the Kaplan–Meier method were drawn for sociodemographic (sex and age at presentation) and clinical variables (e.g., number of presenting signs, congenital microcephaly, cardiac involvement). Death was considered the event of interest. The log‐rank test was used to compare different survival curves. Incidence rates of events were calculated for each category defined by demographic and clinical variables and reported with their 95% confidence intervals. All statistical tests were two‐sided and a *p* < .05 was considered statistically significant. Statistica (release 9.1; StatSoft Corporation) was used for all the bivariate analyses. Stata (release 11.0) was used for the Fisher's exact test and to calculate incidence rates and their 95% confidence intervals.

## 3 RESULTS

We identified 28 new patients harboring biallelic *ITPA* variants (Table S1) and reviewed 12 previously reported subjects with DEE 35 from four peer‐reviewed articles (Bierau et al., 2007; Burgess et al., 2019; Burgis, 2016; Handley et al., 2019; Kaur et al., 2019; Kevelam et al., 2015; Rochtus et al., 2020; Sakamoto et al., 2020), for a total cohort of 40 affected individuals.

### 3.1 *ITPA* Variants

**Figure 1:**
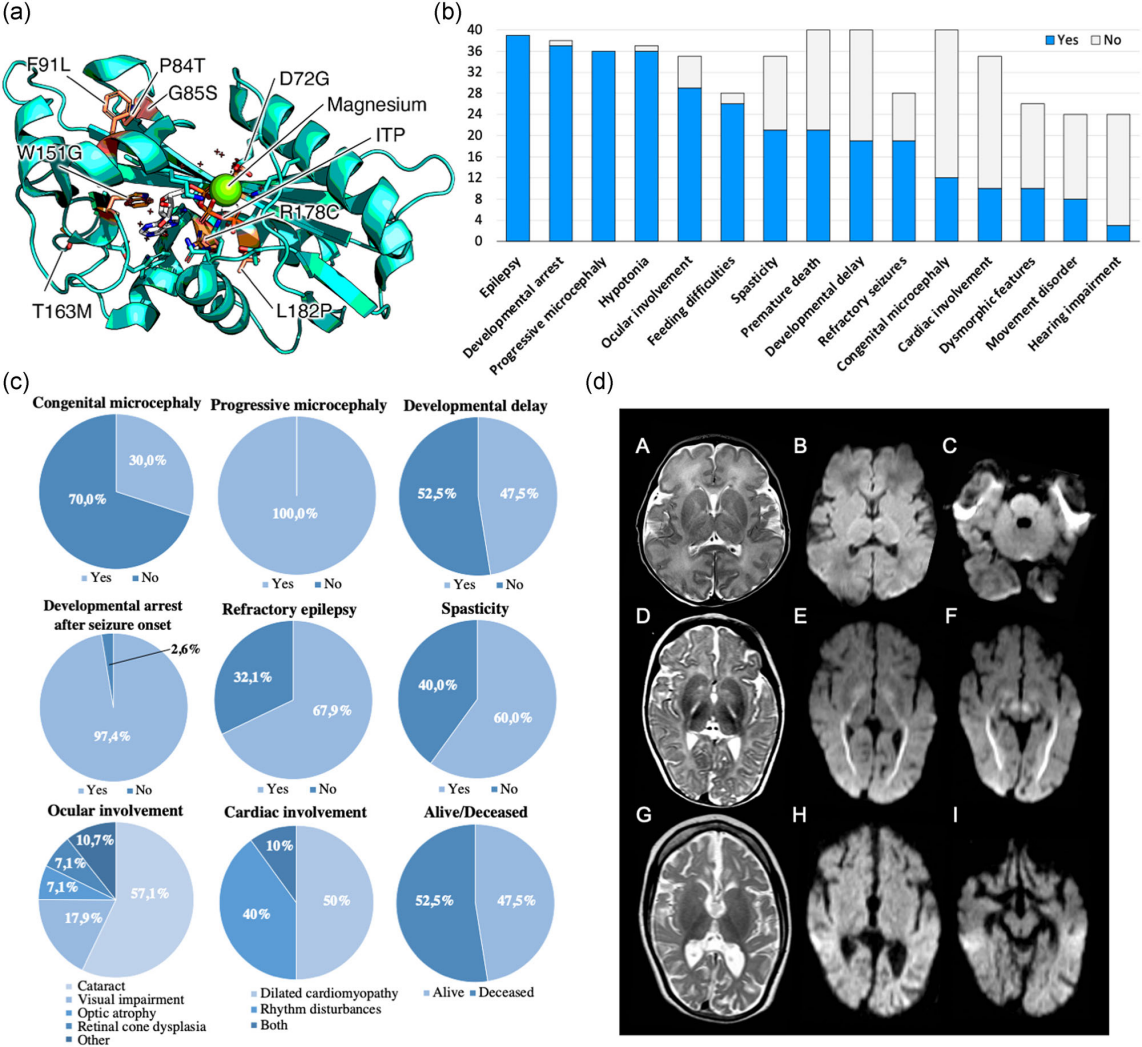
Genetic, clinical, and neuroradiological aspects of DEE 35. (a) Structure model of human ITPA protein showing the localization of the residues affected by *ITPA* missense variants in relation to the ITP‐binding cleft and Mg^2+^ binding site. (b) Bar graph illustrating the distribution of core clinical features of DEE 35, from the most to the less common. Blue bars indicate the number of patients in whom a specific feature is present whereas grey bars indicate the number of subjects in whom that feature was ascertained but it was absent. Ocular involvement includes cataract, visual impairment, optic atrophy, and retinal cone dysplasia. Cardiac involvement consists of dilated cardiomyopathy and rhythm disturbances. Movement disorders include tremors, dystonia, choreoathetoid movements, and dyskinesia. Dysmorphic features were observed in absence of a distinctive facial gestalt. (c) Pie charts illustrating the percent distribution of specific neurological and extra‐neurological manifestations of DEE 35. Rhythm disturbances include tachycardia and long QT syndrome. (d) MRI findings. MRI of P3 at age 6 days (A, B, C). T2‐weighted image (A) shows no atrophy and no signal abnormalities. There is no restricted diffusion (B,C) on diffusion‐weighted imaging (DWI, apparent‐diffusion coefficient maps not shown). MRI of P1 at age 6 months (D, E, F) shows no atrophy, but moderately delayed myelination and T2‐hyperintensity of the posterior limb of the internal capsule (PLIC; D). Restricted diffusion is seen in the optic radiation, PLIC (E), and decussation of the superior cerebellar peduncles (F). Mild diffusion restriction is seen in the globus pallidus (E). MRI of P1 at age 2 years and 8 months (G, H, I) shows seriously deficient myelination and severe cerebral atrophy (G). Restricted diffusion is no longer present (H, I). DEE 35, developmental and epileptic encephalopathy 35; ITP, inosine triphosphate; MRI, magnetic resonance imaging.

Eighteen pathogenic or likely pathogenic variants in *ITPA* were detected in the studied cohort (Table 1). In addition to previously reported variants, 14 novel *ITPA* variants were detected in our cohort, including six missense (Figure 1a; https://michelanglo.sgc.ox.ac.uk/r/itpa) and eight loss‐of‐function (LoF) variants. All these variants were absent in homozygous state from gnomAD, had a low allele frequency in heterozygous state (ranging from 0 to 0.00003551), affected conserved residues, and were predicted to be damaging by several in silico tools (Table 1). In particular, a significant structural destabilization could be predicted for the tested missense variants identified in our cohort as compared to variants frequently found in the healthy population in gnomAD v3.1 (Figure S1). P2 and P3 had a large heterozygous 1.1‐Mb deletion, encompassing *ITPA* (hg19, chr20: 2,816,108–3,955,033), whereas an intragenic deletion, encompassing exons 1–5 (hg19, chr20: 3,189,364–3,196,608) was detected in P27 (Supporting Information Material). Seventeen subjects carried homozygous *ITPA* variants, whereas compound heterozygous variants were found in the remaining individuals. Sanger sequencing confirmed a carrier status for all the parents and showed concordant segregation of the variants with the clinical phenotype.

**Table 1:** Molecular spectrum of *ITPA* variants

*ITPA* variant [NM_033453.3]	g. (hg19)	Source	In‐house database	ExAC/gnomAD	GME; IR	ClinVar (ID)/dbSNP/PMID	SIFT	Mutation taster	HSF	GERP score	CADD score	ACMG/AMP classification	Min. distance	∆∆*G* (kcal/mol)
c.67‐1G>A; p.?	g.3193814 G>A	This study	‐	‐	‐; ‐	‐	N/A	DC (1)	Broken WT Acceptor Site^a^	5.49	34	P (PVS1, PM2, PP3)	‐	‐
c.124+1G>A; p.?	g.3193873 G>A	This study	‐	‐	‐; ‐	LP (VCV000646228.2)	N/A	DC (1)	Broken WT Donor Site	5.45	33	P (PVS1, PM2, PP3, PP5)	‐	‐
c.124+1G>C; p.?	g.3193873 G>C	This study	‐	0.000007952 (2 het)	‐; ‐	rs376142053	N/A	DC (1)	Broken WT Donor Site	5.45	33	P (PVS1, PM2, PP3)	‐	‐
c.142delG; p.(Glu48Serfs*41)	g.3193979 delG	This study	‐	‐	‐; ‐	‐	N/A	DC (1)	N/A	5.68	N/A	P (PVS1, PM2, PP3)	‐	‐
c.215A>G; p.(Asp72Gly)	g.3194656 A>G	This study	‐	‐	‐; ‐	‐	D (0)	DC (1)	N/A	5.32	33	LP (PS4, PM2, PP1, PP3, PP4)	3.0^b^	+4.5
c.250C>A; p.Pro84Thr	g.3194691 C>A	This study	‐	‐	‐; ‐	‐	D (0)	DC (1)	N/A	5.32	26.8	VUS (PM2, PP3, PP4)	7.2	+6.9
c.253G>A; p.Gly85Ser	g.3194694 G>A	This study	‐	0.000003977 (1 het)	‐; ‐	rs1343080275	D (0)	DC (1)	N/A	5.32	28.5	VUS (PM2, PP3, PP4)	5.2	+48 (approx.)
c.264‐1G>A p.(Ile88Metfs*59)	g.3195926 G>A	Sakamoto et al. (2020); this study	‐	0.000007954 (2 het)	‐; ‐	rs781254071	N/A	DC (1)	Broken WT Acceptor Site	5.77	34	P (PVS1, PM2, PP3)	‐	‐
c.264‐607_295+1267del p.?	g.3195320 del	Kevelam et al. (2015)	‐	‐	‐; ‐	P (VCV000218088)/26224535	N/A	N/A	Splice junction loss	N/A	N/A	P (PVS1, PM2, PP3)	‐	‐
c.271T>C p.(Phe91Leu)	g.3195934 T>C	This study	‐	‐	‐; ‐	LP (VCV000807614)	D (0)	DC (1)	N/A	5.77	28.9	LP (PM2, PM3, PP3, PP5)	7.3	+7.0
c.359_366dup TCAGCACC p.(Gly123Serfs*104)	g.3199225 dupCTCAGCAC	Kevelam et al. (2015); this study	‐	0.00003551 (10 het)	‐; ‐	LP (SCV001168653)/rs946985349/26224535	N/A	N/A	N/A	N/A	N/A	P (PVS1, PM2, PP3, PP5)	‐	‐
c.451T>G p.(Trp151Gly)	g.3202526 T>G	Kaur et al. (2019)	‐	‐	‐; ‐	‐	D (0)	DC (1)	N/A	5.32	32	LP (PM1, PM2, PP3, PP4, PP5)	2.3	+12.5
c.452G>A p.(Trp151*)	g.3202527 G>A	Kevelam et al.; this study (2015)	‐	0.00005309 (15 het)	‐; ‐	P (VCV000218089)/rs200086262/26224535, 30856165, 31618474	D due to stop	DC (1)	N/A	5.32	46	P (PVS1, PM2, PP3)	‐	‐
c.456_488+7del p.?	g.3202528 del	Handley et al. (2019)	‐	‐	‐; ‐	‐	N/A	N/A	Splice junction loss	N/A	N/A	P (PVS1, PM2, PP3)	‐	‐
c.488C>T p.(Thr163Met)	g.3202563 C>T	This study	‐	0.000007970 (2 het)	0.000503525 (1 het); ‐	VUS (VCV000431714)/rs758706191	D (0)	DC (1)	N/A	5.32	34	LP (PM1, PM2, PP3, PP4)	8.1	+6.1
c.488+5_488+6delGG p.?	g.3202568 delGG	This study	‐	‐	‐; ‐	‐	N/A	N/A	Broken WT Donor Site	5.32	N/A	LP (PM2, PP3, PP4)	‐	‐
c.489‐2A>G; p.?	g.3204010 A>G	This study	‐	‐	‐; ‐	‐	N/A	DC (1)	Broken WT Acceptor Site	5.82	34	P (PVS1, PM2, PP3)	‐	‐
c.489‐1 G>A; p.?	g.3204011 G>A	Sakamoto et al. (2020)	‐	‐	‐; ‐	‐	N/A	DC (1)	Broken WT Acceptor Site	5.82	34	P (PVS1, PM2, PP3)	‐	‐
c.489‐1 G>T; p.?	g.3204011 G>T	This study	‐	‐	‐; ‐	‐	N/A	DC (1)	Broken WT Acceptor Site	5.82	34	P (PVS1, PM2, PP3)	‐	‐
c.519delC; p.(Asn173Lysfs*51)	g.3204042 delC	This study	‐	0.00001595 (4 het)	‐; ‐	rs748042110	N/A	DC (1)	N/A	5.82	N/A	P (PVS1, PM2, PP4)	‐	‐
c.532C>T; p.Arg178Cys	g.3204055 C>T	Kevelam et al. (2015); this study	‐	0.000003988 (1 het)	‐; ‐	P (VCV000218090)/rs746930990/26224535	D (0)	DC (1)	N/A	5.82	32	LP (PM1, PM2, PP3, PP4, PP5)	1.9	+5.0
c.545T>C; p.Leu182Pro	g.3204068 T>C	This study	‐	‐	‐; ‐	VUS (VCV000452647)	D (0)	DC (1)	N/A	5.82	26.4	VUS (PM2, PP3, PP4)	9.4	33 (approx.)

Abbreviations: ACMG/AMP, American College of Medical Genetics and Genomics and the Association for Molecular Pathology; CADD, Combined Annotation Dependent Depletion; ∆∆*G*, difference in relative Gibbs free energy of folding; D, damaging; DC, disease‐causing, GERP, Genomic Evolutionary Rate Profiling; GME, Greater Middle East Variome Project; HSF, human splice finder; IR, Iranome; LP, likely pathogenic; Min., distance closest distance of a residue atom to either inosine triphosphate (ITP) or Mg2+ in the model; N/A, not applicable; P, pathogenic; SIFT, Sorting Intolerant From Tolerant; SNP, single‐nucleotide polymorphism; VUS variant of unknown significance; WT, wildtype.

^a^ Possible additional activation of an intronic cryptic acceptor site.

^b^ Probable catalytic residue. Variants reported according to RefSeq NM_033453.3, GenBank NC_000020.11.

### 3.2 Clinical delineation of DEE 35

The phenotype observed in the studied cohort was consistent with severe DEE (Figure 1b). The age range (current age) was 1–72 months, with a median age at presentation of 3 months, the median age at last follow‐up of 42 months, and a male‐to‐female sex ratio of 0.69. Severe DD in the first few months of life was diagnosed in 19/40 (47.5%) patients, whereas developmental stagnation/arrest after seizure onset was present in all subjects (Table 2). A variable number (1–4) of presenting signs was observed (Table 2). In particular, congenital microcephaly was diagnosed in 30% of cases. Interestingly, poor neonatal adaptation was only occasionally observed (P2 and P3). Approximately one‐fifth of subjects were born small for gestational age (SGA) and significant swallowing difficulties were very common from birth onwards (92.9%), leading to failure to thrive in all cases. The age at first seizure ranged from 2 days to 7 months, with a median age of 4 months. Febrile seizures were only observed in one case (P15), at the age of 4 months. All affected individuals had epilepsy, with refractory seizures in 68% of patients (Figure 1c). Clonic/myoclonic, tonic, and tonic‐clonic seizures were observed, occasionally leading to status epilepticus in two patients (P4 and P9). Electroencephalographic abnormalities were variable and included focal, multifocal, and diffuse/generalized discharges often within a slow and disorganized background, consistent with the underlying encephalopathy (Table S1).

**Table 2:** Clinical features of DEE 35 patients

	***n*/*N* (%)**
*Characteristics at clinical presentation*	
Sex	
Male	12/38 (31.6)
Female	26/38 (68.4)
Age at presentation (months), median [1st–3rd quartile]	3 [1 − 4]
Age at first seizure (months), median [1st–3rd quartile]	4 [2 − 5]
No. of presenting signs^a^	
1	15/40 (37.5)
2	20/40 (50.0)
3	3/40 (7.5)
4	2/40 (5.0)
Congenital microcephaly	12/40 (30.0)
Developmental delay	19/40 (47.5)
Seizures	27/40 (67.5)
Small for gestational age	7/34 (20.6)
*Clinical features*	
Progressive microcephaly	36/36 (100)
Epilepsy	39/39 (100)
Refractory seizures	19/28 (67.9)
Developmental arrest after seizure onset	37/38 (97.4)
Feeding difficulties	26/28 (92.9)
Progressive hypotonia	36/37 (97.3)
Spasticity	21/35 (60.0)
Movement disorder	8/24 (33.3)
Ocular involvement	29/35 (82.9)
Cataract	16/28 (57.1)
Visual impairment	5/28 (17.9)
Optic atrophy	2/28 (7.1)
Retinal cone dysplasia	2/28 (7.1)
Other^b^	3/28 (10.7)
Cardiac involvement	10/35 (28.6)
Dilated cardiomyopathy	5/10 (50.0)
Rhythm disturbances	4/10 (40.0)
Both	1/10 (10.0)
Dysmorphic features	10/26 (38.5)
Hearing impairment	3/24 (12.5)
Life status	
Alive	19/40 (47.5)
Death	21/40 (52.5)

Abbreviations: DEE 35, developmental and epileptic encephalopathy 35; *N*, number.

^a^ Including microcephaly, psychomotor delay, seizures, hypotonia, movement disorder.

^b^ Strabismus and refractive errors.

Progressive microcephaly and axial hypotonia were common, occurring in 100% and 97.3% of patients, respectively. Additional relevant neurological findings included appendicular spasticity with hyperreflexia (60%) and hyperkinetic movement disorders (33%) (e.g., tremors, choreoathetoid movements, dystonia, and dyskinesia). Extraneurological manifestations were frequent and consisted of variable ocular disorders in 83% of cases and cardiomyopathy and/or rhythm disturbances in 29% of patients. Mild dysmorphic features were present in 39% of subjects, but a definite facial gestalt is absent. Early death occurred in 53% of cases, with a median age at death of 24 months. Causes of death included cardiac dysfunction in subjects with heart disease and seizure‐related apnoea (P2 and P24) or aspiration pneumonia (P17 and P18) in those without cardiac involvement.

### 3.3 Neuroradiological phenotype

Twenty brain MRI scans (of 13 patients) were of sufficient quality to be analyzed in detail (Figure 1d). MRI characteristics per age group are reported in Table 3. In patients younger than 2 months (three scans in three patients), brain MRI revealed very few abnormalities. In particular, myelination was normal and no cerebral or cerebellar atrophy or other brain lesions were observed. The posterior limb of the internal capsule (PLIC) did not show the normal low T2 signal in one scan, but no focal lesion was present. DWI was available in two MRI scans and one showed restricted diffusion of specific structures (Table S2). In patients aged between 2 and 4 months (four scans in three patients), only one scan showed slightly delayed myelination. The PLIC contained a T2‐hyperintense focal lesion in two scans. Initial cerebral atrophy could be observed in all scans and cerebellar atrophy was present in one scan. DWI was available in three MRI scans and restricted diffusion of specific structures was present in two (Table S2). In patients aged between 4 and 8 months (7 scans in 7 patients), variably delayed myelination was present in six scans. The PLIC contained a T2‐hyperintense lesion in six scans. Mild‐to‐moderate cerebral atrophy was seen in five scans, typically associated with a thin corpus callosum, while no cerebellar atrophy was observed. In all scans, there was restricted diffusion of specific structures (Table S3). In patients older than 8 months (six scans in four patients), all scans showed moderately to severely delayed myelination (Table S4). The PLIC contained a T2‐hyperintense lesion in three scans. The thalamus was atrophic in two scans. There was mild‐to‐severe cerebral atrophy with a thin corpus callosum in all, and mild‐to‐moderate cerebellar atrophy in four scans. Restricted diffusion of specific structures was present in three patients (Table S4).

**Table 3:** Brain MRI characteristics per age

Age at MRI (months)	≤2	2 to ≤4	4 to ≤8	>8
Number of patients/scans	3 patients/3 scans	3 patients/4 scans	7 patients/7 scans	4 patients/6 scans
Myelination: delayed	0/3	1/4 slightly delayed	2/7 slightly delayed, 2/7 mildly delayed, 2/7 delayed	4/6 delayed, 2/6 severely delayed
Cerebral cortex: abn. T2 signal	0/3	0/4	0/7	0/6
Cerebral hemispheric WM: abn. T2 signal	0/3	0/4	0/7	0/6
Basal nuclei/thalami: abn. T2 signal	0/3	0/4	1/7, globus pallidus	1/6, globus pallidus
ALIC: abn. T2 signal	0/3	0/4	1/7	0/6
PLIC: abn. T2 signal	1/3, no low signal	2/4	6/7	3/6
Midbrain: abn. T2 signal	0/3	0/4	1/7, decussation SCP and left crus cerebri	1/6, WM around red nucleus
Pons: abn. T2 signal	0/3	1/4, CTT	0/7	1/6, CTT
Medulla: abn. T2 signal	0/3	0/4	0/7	1/6, everything except inf. olivary n.
SCP: abn. T2 signal	0/3	0/4	0/7	0/6
MCP: abn. T2 signal	0/3	0/4	2/7	1/6
Cerebellar cortex: abn. T2 signal	0/3	0/4	0/7	0/6
Cerebellar WM: abn. T2 signal	0/3	1/4	1/7	1/6, peridentate WM
Hilus dentate n.: abn. T2 signal	0/3	0/4	1/7	0/6
Cerebral atrophy	0/3	3/4 slight, 1/4 mild	3/7 mild, 2/7 moderate	1/6 mild, 1/6 moderate, 3/6 moderate‐severe, 1/6 severe
Corpus callosum: thin	0/3	0/4	4/7	6/6
Thalamus: atrophy	0/3	0/4	0/7	2/6
Cerebellar atrophy	0/3	1/4 slight	0/7	1/6 slight, 2/6 mild, 1/6 moderate
Diffusion restriction	1/2	2/3	5/5	3/5
Diffusion restriction specified	globus pallidus, PLIC, left crus cerebri, decussation SCP, SCP, hilus dentate n., cerebellar WM, MCP, ICP	OR 1/2, globus pallidus 1/2, PLIC 2/2, crus cerebri 1/2, brachium inf. colliculus 1/2, CTT in midbrain, pons, and medulla 1/2, decussation SCP 1/2, SCP 1/2, hilus dentate n. 1/2	OR 4/5, globus pallidus 4/5, ALIC 1/5, PLIC 5/5, crus cerebri 4/5, brachium inf. colliculus 2/5, CTT in pons 2/5, decussation SCP 5/5, SCP 4/5, hilus dentate n. 1/5, cerebellar WM 2/5, ICP 2/5, pyramids 1/5	cerebral hemispheric WM 2/3, corpus callosum 1/3, OR 2/3, ALIC 3/3, PLIC 3/3, crus cerebri 1/3, WM around red nucleus 1/3, brachium inf. colliculus 1/3, CTT in pons 2/3, decussation SCP 2/3, SCP 1/3, cerebellar WM 1/5, MCP 1/3, ICP 2/3
Contrast abnormalities	0/0	0/0	0/3	0/1
MR spectroscopy: lactate elevation	0/1	0/2	1/1	0/2
Extra features	1/3 dilated inferior horns, 1/3 rarefaction of temporal poles	0/4	2/7 perivascular spaces, 1/7 PLIC and left crus cerebri rarefied	1/6 rarefaction of temporal poles

Abbreviations: Abn., abnormal; ALIC, anterior limb of the internal capsule; CTT, central tegmental tracts; ICP, inferior cerebellar peduncle; MCP, middle cerebellar peduncle; MRI, magnetic resonance imaging; n, nucleus; OR, optic radiation; PLIC, posterior limb of the internal capsule; SCP, superior cerebellar peduncle; WM, white matter.

Diffusion restriction was separately reviewed. Commonly involved structures were globus pallidus, PLIC, pyramidal tracts in the brain stem, cerebellar white matter, hilus of the dentate nucleus, superior cerebellar peduncles, decussation of the superior cerebellar peduncles, middle cerebellar peduncles, optic radiation, brachium of the inferior colliculus, and central tegmental tracts in the midbrain, pons, and medulla. In older patients, restricted diffusion could also be observed in the anterior limb of the internal capsule, corpus callosum, and cerebral hemispheric white matter.

Two patients had sequential MRI scans. In one case, restricted diffusion was absent at 2.5 months but present in specific structures at 4 months. The other patient had four MRI scans (at 6 months, 1 year, 1.7 years, and 2.8 years). Restricted diffusion of specific structures and a T2‐hyperintense lesion of the PLIC were present at 6 months and 1 year but were not observed at 1.7 and 2.8 years. Cerebral and cerebellar atrophy increased over time.

### 3.4 Predictors of early mortality in DEE 35

**Figure 2:**
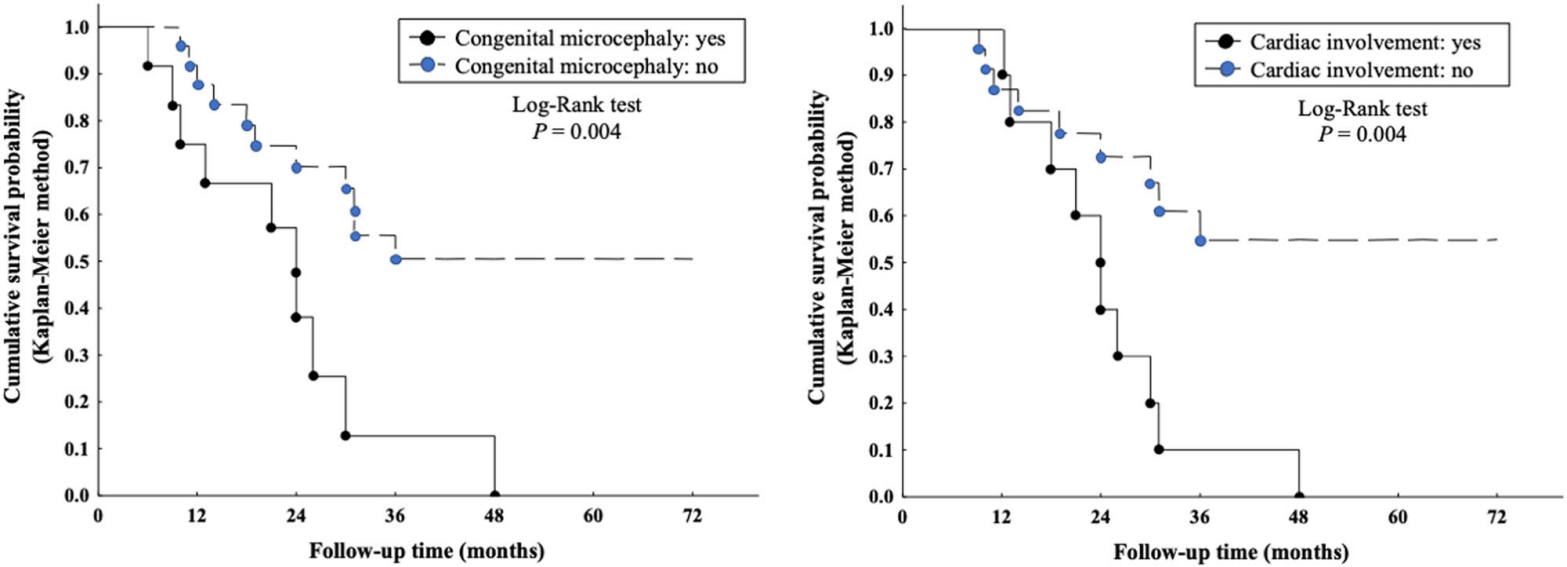
Outcome predictors in developmental and epileptic encephalopathy 35. Survival curves according to the Kaplan–Meier method to the presence/absence of congenital microcephaly and cardiac involvement. Congenital microcephaly and cardiac involvement are independent clinical prognostic factors of poor outcome (*p* = .004)

Epidemiologic and clinical parameters were considered for the investigation of outcome predictors through the analysis of number/percentage and incidence of death events (Table 4). The studied categories included sex, age at presentation, age at first seizure, number of presenting signs, congenital microcephaly, DD, seizures, hypotonia, SGA status, spasticity, ocular involvement, and cardiac involvement. Among these, congenital microcephaly and cardiac disorders were significantly associated with poor disease outcomes (*p* = .004) (Figure 2). In fact, 10/12 (83%) patients with congenital microcephaly prematurely deceased versus 11/28 (39.3%) subjects with normal occipitofrontal circumference (OFC) at birth. The Incidence Rate (IR) of subjects with congenital microcephaly was 4.032 (95% CI = 2.17–7.494) per 100 person‐months, while it was 1.279 (95% CI = 0.708−2.31) in those with normal OFC at birth. The Hazard Ratio (HR) was 3.427 (95% CI = 1.402−8.373). This supports a positive relationship (*p* = .004) between the presence of this clinical feature and premature death. Similarly, early lethality was observed in 10/10 (100%) subjects with some type of cardiac involvement versus 9/25 (36%) lacking cardiac abnormalities. The IR among subjects with cardiac manifestations was 4.049; (95% CI = 2.178−7.525) versus 1.155 (95% CI = 0.601−2.220) in those lacking cardiac involvement. The HR was 3.509 (95% CI = 1.394−8.834). These findings are suggestive of a higher mortality rate among affected individuals with cardiac disorders (*p* = .004). No other statistically significant associations were detected for the remaining variables (Table 4).

**Table 4:** Number and percentage of events and incidence rates of deaths by different clinical categories

	No. of deaths/No. of patients (%)	*p*	Incidence rate × 100 persons‐month (95% CI)	*p* (Log‐rank test)
All patients	21/38 (55.3)		1.895 (1.236−2.907)	
Sex				
Male	4/12 (33.3)	.16*	1.031 (0.387−2.747)	.27
Female	15/26 (57.7)		2.137 (1.288−3.544)	
Age at presentation (months)				
<3	6/17 (35.3)	.10*	1.382 (0.621−3.077)	.38
≥3	12/19 (63.2)		2.19 (1.244−3.856)	
Age at first seizures (months)				
<4	7/15 (46.7)	.85*	1.877 (0.895−3.937)	.54
≥4	8/16 (50)		1.509 (0.755−3.018)	
No. of presenting signs				
1	6/15 (40)	.38**	1.307 (0.587−2.909)	.19
2	11/20 (55)		2 (1.108−3.611)	
3–4	4/5 (80)		4.04 (0.516−0.765)	
Congenital microcephaly				
Yes	10/12 (83.3)	**.011** *	4.032 (2.17−7.494)	**.004**
No	11/28 (39.3)		1.279 (0.708−2.3)	
Developmental delay				
Yes	12/19 (63.2)	.20*	2.065 (1.173−3.637)	.71
No	9/21 (42.9)		1.708 (0.889−3.282)	
Seizures				
Yes	14/27 (51.9)	1.00**	1.889 (1.119−3.19)	.95
No	7/13 (53.8)		1.907 (0.909−4)	
Hypotonia				
Yes	2/6 (33.3)	.40**	2.439 (0.61−9.752)	.60
No	19/34 (55.9)		1.851 (1.181−2.903)	
Small for gestational age				
Yes	3/7 (42.9)	.68**	1.714 (0.553−5.315)	.95
No	15/27 (55.6)		1.923 (1.159−3.19)	
Spasticity				
Yes	12/21 (57.1)	.10*	2.194 (1.246−3.863)	.13
No	4/14 (28.6)		0.926 (0.348−2.467)	
Ocular involvement				
Yes	17/29 (58.6)	.63**	1.959 (1.218−3.151)	.96
No	2/6 (33.3)		1.835 (0.459−7.336)	
Cardiac involvement				
Yes	10/10 (100)	**.001** **	4.049 (2.178−7.525)	**.004**
No	9/25 (36)		1.155 (0.601−2.22)	

*Note*: 95% CI is the 95% confidence interval of the incidence rate.

* *p* Fisher's exact test.

^**^ *p* Pearson's *χ* ^2^.

## 4 DISCUSSION

This is the largest study involving subjects with DEE 35 harboring (likely) pathogenic variants in *ITPA*, allowing to delineate the molecular spectrum, determine the clinical phenotype, identify the neuroimaging patterns, and eventually establish prognostic factors in this rare and severe neurological condition.

### 4.1 ITPase deficiency

ITPase is a pyrophosphohydrolase catalyzing the conversion of noncanonical purines (NCPs) into the corresponding nucleoside monophosphate (Simone et al., 2013). The 45‐kDa enzyme has a homodimeric structure composed of two globular 194‐aminoacid α/β structural elements supported by a central elongated mixed β‐sheet (Behmanesh et al., 2009; Holmes et al., 1979). The ITP‐binding cleft is located between the dimerization and the N‐terminal lobes, whereas the Mg^2+^‐dependent and pH‐sensitive catalytic activity lie in a region of the N‐terminal lobe close to the dimer interface (Behmanesh et al., 2009; Holmes et al., 1979). NCPs may spontaneously originate from the purine biosynthesis pathway or the deamination of nucleosides and nucleotides containing adenine or guanine (Abolhassani et al., 2010; Galperin et al., 2006). The increased sensitivity to NCPs caused by ITPase deficiency may lead to delayed cell cycle progression, increased mutation rate, and DNA damage, supporting a pivotal role of ITPase‐mediated genomic instability limitation in cellular homeostasis (Abolhassani et al., 2010; Holmes et al., 1979). An involvement of ITPase in immunity and drug metabolism has also been reported (Nakauchi et al., 2016; Shipkova et al., 2011).

*ITPA* is highly expressed in the central nervous system (especially in neurons) and the heart (Holmes et al., 1979). The accumulation of NCPs resulting from ITPase deficiency can cause direct cellular toxicity, eventually leading to neuronal apoptosis (Kevelam et al., 2015). Additionally, the NPCs excess may negatively affect the function of enzymes utilizing adenosine triphosphate (ATP) or guanosine triphosphate, representing an indirect mechanism of neuronal toxicity. Indeed, the alteration of G‐protein signal transduction leads to the inappropriate regulation of critical neuronal processes (e.g., neurotransmitter release, neuronal plasticity, and glucose metabolism) (Kevelam et al., 2015). Accordingly, ITPase deficiency resulting from biallelic *ITPA* pathogenic variants cause a severe DEE with progressive disease course and neuroimaging abnormalities.

### 4.2 Spectrum of *ITPA* variants

Hitherto, 24 distinct *ITPA* variants are known to be associated with DEE 35, including 19 single‐nucleotide variants (SNVs), three intragenic deletions, one intragenic duplication, and one whole gene deletion within a larger chromosomal rearrangement. In our cohort, four out of the eight previously reported pathogenic variants were detected: c.264‐1G>A; p.(Ile88Metfs*59) (Sakamoto et al., 2020), c.359_366dup; p.(Gly123Serfs*104) (Kevelam et al., 2015), c.452G>A;p.Trp151* (Kevelam et al., 2015), c.489‐1G>A (Sakamoto et al., 2020). We additionally identified 14 novel SNVs, all rare (allele frequency < 0.001), affecting conserved residues (GERP score range 5.32–5.82), and predicted damaging by in silico tools (CADD score range 26.4–46, ∆∆*G* all greater than 5 kcal/mol) (Table 1). Splicing and frameshift variants likely lead to truncated transcripts or nonsense‐mediated decay. Missense variants are predicted to alter the structure of the ITP‐binding cleft, interfere with the Mg^2+^‐dependent catalytic activity, and/or impair dimerization, eventually leading to a deficient enzymatic function. A loss of function mechanism is also expected in patients harboring partial or whole gene deletions. This is in line with what is usually observed in several epileptic disorders due to underlying metabolic deficiency and offers the possibility of an etiology‐specific treatment (Assi et al., 2017; Rahman et al., 2013; Sharma & Prasad, 2017).

We detected a multiexon deletion (P27) involving exons 1–5 of *ITPA* and a whole gene deletion (P2 and P3) in the context of a 1.1‐Mb deletion, additionally involving *AVP*, *DDRGK1*, *PANK2*, and *SLC4A11*. Biallelic variants in *DDRGK1*, *PANK2*, and *SLC4A11* cause variable clinical conditions (Supporting Information Results), whereas *AVP* (MIM# 192340) and *SLC4A11* (MIM# 610206) haploinsufficiency is associated with autosomal dominant neurohypophyseal diabetes insipidus (MIM# 125700) and corneal dystrophy (Fuchs endothelial, type 4; MIM# 613268) (Christensen et al., 2004; Vithana et al., 2008). However, the two subjects harboring the deletion did not display features suggestive of these conditions. In line with these observations, all the detected *ITPA* are predicted to result in ITPase deficiency, supporting an underlying LoF pathogenic model in DEE 35.

### 4.3 Phenotypic spectrum of DEE 35

Affected individuals present with a severe DEE, in which the underlying metabolic defect is responsible for the absence of development and the uncontrolled epileptic activity additionally contributes to worsen the cognitive impairment (Kevelam et al., 2015; McTague et al., 2016). Although only a portion of patients presents with DD before seizure onset (48%), microcephaly (congenital, 30%), and seizures (67.5%) in the earliest stages of the disorder (Table 2), most will develop the neurological hallmarks of DEE as the disease progresses. The cardinal clinical features of DEE 35, reported in >90% of cases, include progressive microcephaly (100%), epilepsy (100%), developmental stagnation after seizure onset (97.4%), progressive hypotonia (97.3%), and spasticity (60%). Interestingly, the perinatal period and birth weight are normal in most subjects, whereas neurological involvement and failure to thrive become evident in the first few months of life. Extraneurological manifestations are particularly relevant in, as emerged from the systematic analysis of new cases and the review of previously published patients. A significant subset of subjects presents with ocular and cardiac involvement, which should be considered in all respects as part of the core clinical phenotype and assessed in all cases. Ophthalmic manifestations are present in a large number of affected individuals (83%), including cataract, visual impairment, optic atrophy, and retinal cone dysplasia. Although cardiac disorders are less common (29%), when present they suggest an unfavorable outcome (Figure 2) and primarily contribute to the increased likelihood of early lethality (53% of cases). Accordingly, cardiac involvement was absent in the subjects who survived at 48‐60 months (P1, P4, P5, P6, P13, P15, P19, and P21), although these patients did not show any peculiar genetic or clinical feature as compared to the rest of the cohort (Table S1).

### 4.4 Epileptic phenotype

Epilepsy is the cardinal feature of DEE 35, being observed in all affected individuals (Table 2). The epileptic phenotype mainly consists of focal and multifocal clonic/myoclonic seizures, generalized tonic seizures, and generalized tonic‐clonic seizures. Patients suffer from both afebrile and, less often, febrile seizures. Status epilepticus may also occur (such as in P4 and P9). EEG usually shows focal or multifocal epileptiform discharges in the context of a slowing and disorganization of the background cerebral activity, consistent with the underlying encephalopathy. Several different ASMs have been employed alone or in combination (e.g., clonazepam, levetiracetam, topiramate, phenytoin, phenobarbitone, vigabatrin, and clobazam), but the response to therapy is usually poor, and refractory seizures occur in more than two‐thirds of cases.

Ketogenic diet (KD), a low‐carbohydrate dietary regimen reducing neuronal excitability, has proven to be effective in the management of refractory epilepsy in children with DEEs (Jagadish et al., 2019; Martin et al., 2016; Wells et al., 2020). More specifically, through several mechanisms working in concert (anti‐inflammatory activity, epigenetic function, restoration of bioenergetics, synaptic dysfunction, and impaired redox homeostasis) KD has been successfully used in many epileptic disorders caused by an underlying metabolic deficiency (Gavrilovici & Rho, 2021; Lin Lin Lee et al., 2018). Among these, KD proved beneficial in metabolic epilepsies involving dysfunctional energy utilization (e.g., glucose transporter type 1 deficiency syndrome (GLUT1‐DS) and pyruvate dehydrogenase complex deficiency) or abnormal neurotransmitter degradation (succinic semialdehyde dehydrogenase deficiency and non‐ketotic hyperglycinemia), as well as in mitochondrial epilepsies (e.g., *POLG*‐related disorders and Leigh syndrome) (Gavrilovici & Rho, 2021; Lin Lin Lee et al., 2018). In our cohort, KD was administered in four cases (P2, P4, P9, and P12). Although this approach was apparently ineffective in P2, a better seizure control (decreased seizure frequency) was temporarily achieved in P9 and P12, whereas the efficacy is still under investigation in P4. Interestingly, KD may directly increase ATP and adenosine levels, both recognized as crucial modulators of epileptogenic activity (Boison, 2017; Gavrilovici & Rho, 2021; Masino et al., 2010). In principle, this effect might prove useful in the biochemical context of ITPase deficiency (Burgis, 2016; Masino et al., 2010). However, further dedicated studies are necessary to confirm this hypothesis and investigate whether KD may be considered a potential treatment option in DEE 35.

### 4.5 Neuroimaging spectrum

In the very early stages of the disease, MRI may not reveal abnormalities. The most typical feature of DEE 35, which appears after a few months, is a narrow, short segment of T2 hyperintensity in the PLIC, which disappears after several months (Table 3). Over time, delayed myelination becomes progressively more evident. DWI is most useful, revealing the involvement of structures that are not T2 hyperintense. The involved structures are those that typically myelinate early. Only in older patients, diffusion restriction in the anterior limb of the internal capsule and cerebral hemispheric white matter is observed, structures that myelinate later than the PLIC, brain stem, cerebellar white matter, and optic radiation. The globus pallidus and thalamus are the gray matter structures with the highest myelin content and they are the only gray matter structures showing abnormalities. Atrophy is a relatively late finding and increases over time. Cerebral atrophy is earlier and more pronounced than cerebellar atrophy. These peculiar aspects are suggestive of a primary neuronal degeneration associated with Wallerian degeneration of white matter tracts, active Wallerian degeneration being characterized by diffusion restriction (Kevelam et al., 2015). Since early‐myelinating white matter structures are the first to become functionally active, they are precociously involved in the course of the disease (Kevelam et al., 2015). Although this sequence of MRI characteristics is characteristic of DEE 35, the first MRI abnormalities may occur after the clinical presentation and therefore MRI may initially not be informative. If present, the typical lesions in the posterior limb of the internal capsule and the pattern of structures with diffusion restriction are suggestive for DEE 35 diagnosis and may help differentiate this condition from *RABGAP2*‐related phenotypes (e.g., diffuse brain atrophy, frontotemporal polymicrogyria, and corpus callosum hypoplasia) (Sakamoto et al., 2020).

### 4.6 Cardiac involvement in patients with pathogenic *ITPA* variants

The excess of ITP in cardiac sarcomeres favors the abnormal actomyosin binding of ITP instead of ATP and the accumulation of deoxyinosine monophosphate in nucleic acids (Behmanesh et al., 2009; Burton et al., 2005). These events eventually lead to cardiac toxicity as a result of disorganization of the sarcomeric structure in the developing heart, increased DNA damage, nonfunctional RNAs, delayed cell cycle progression, and impaired cardiac protein function (Behmanesh et al., 2009; Burton et al., 2005). Cardiac involvement is of particular interest in DEE 35 individuals. Lethal infantile‐onset dilated cardiomyopathy was recently reported in two subjects with Martsolf‐like syndrome harboring homozygous null *ITPA* variants (Handley et al., 2019). In this study, cardiomyopathy (5/10 subjects), rhythm disturbances (4/10 subjects), or both (1/10 subjects) were observed in 29% of patients (Table 2), making cardiac involvement a key clinical issue in ITPase deficiency. Although there is still limited direct evidence to unveil the potential mechanisms underlying cardiac dysfunction in human subjects with ITPase deficiency, it is tempting to speculate that the restoration of the enzymatic activity might positively impact on cardiac function in these patients (Burgis, 2016; Handley et al., 2019).

## 5 CONCLUSIONS

Early diagnosis and timely antiepileptic treatment may favorably impact the management and developmental outcomes of patients with DEEs. Obtaining a genetic diagnosis is especially crucial for parental counseling and beneficial in terms of knowledge of the natural course of the specific disorder. We reported a comprehensive analysis of 40 patients with DEE 35, expanding and refining the molecular and phenotypic spectrum of this severe condition. In addition to a severe, progressive encephalopathy which typically presents during the first months of life, affected individuals show a high prevalence of ocular and cardiac manifestations and an increased risk of premature death. Congenital microcephaly and cardiac involvement are independent clinical predictors of poor outcomes. Taken together, these findings may have a large impact on diagnosis, counseling, and follow‐up of subjects with DEE 35.

## CONFLICT OF INTERESTS

Teresa Santiago‐Sim, Amy Dameron, and Hui Yang are employees of GeneDx, Inc. The remaining authors declare that there are no conflict of interests.

## AUTHOR CONTRIBUTIONS

*Conceptualization*: Marcello Scala, Saskia B. Wortmann, Namik Kaya, Menno D. Stellingwerff, Angela Pistorio, Marjo S. van der Knaap, and Reza Maroofian. *Data curation*: Marcello Scala, Saskia B. Wortmann, Namik Kaya, Menno D. Stellingwerff, Angela Pistorio, Marjo S. van der Knaap, and Reza Maroofian. *Formal analysis*: Marcello Scala, Saskia B. Wortmann, Menno D. Stellingwerff, Angela Pistorio, and Marjo S. van der Knaap. *Investigation*: Emma Glamuzina, Clara D van Karnebeek, Cristina Skrypnyk, Katarzyna Iwanicka‐Pronicka, Dorota Piekutowska‐Abramczuk, Elżbieta Ciara, Frederic Tort, Beth Sheidley, Annapurna Poduri, Parul Jayakar, Anuj Jayakar, Jariya Upadia, Nicolette Walano, Tobias B. Haack, Holger Prokisch, Hesham Aldhalaan, Ehsan Ghayoor Karimiani, Yilmaz Yildiz, Ahmet Cevdet Ceylan, Teresa Santiago‐Sim, Amy Damero, Hui Yang, Mehran Beiraghi Toosi, Farah Ashrafzadeh, Javad Akhondian, Shima Imannezhad, Hanieh Sadat Mirzadeh, Shazia Maqbool, Aisha Farid, Mohamed A. Al‐Muhaizea, Meznah Oudah Alshwameen, Lama Aldowsari, Maysoon Alsagob, Ashwaq Alyousef, Rawan AlMass, Aljouhra AlHargan, Ali H. Alwadei, Maha M. AlRasheed, Dilek Colak, Hanan Alqudairy, Sameena Khan, Matthew A. Lines, M. Ángeles García Cazorla, Antonia Ribes, Eva Morava, Farah Bibi, Shahzad Haider, Matteo P. Ferla, Jenny C. Taylor, Hessa S. Alsaif, Abdulwahab Firdous, Mais Hashem, Chingiz Shashkin, Kairgali Koneev, Rauan Kaiyrzhanov, Stephanie Efthymiou, Queen Square Genomics, Thomas Schmitt‐Mechelke, Andreas Ziegler, Mahmoud Y. Issa, Hasnaa M. Elbendary, Pasquale Striano, Fowzan S. Alkuraya, Maha S. Zaki, Joseph G. Gleeson, Tahsin Stefan Barakat, and Jorgen Bierau. *Project administration*: Marjo S. van der Knaap and Henry Houlden. *Visualization*: Marcello Scala, Saskia B. Wortmann, Namik Kaya, Menno D. Stellingwerff, Angela Pistorio, and Marjo S. van der Knaap. *Writing—original draft*: Marcello Scala, Saskia B. Wortmann, Menno D. Stellingwerff, and Marjo S. van der Knaap. *Writing—review & editing*: Marcello Scala, Saskia B. Wortmann, Menno D. Stellingwerff, Pasquale Striano, Marjo S. van der Knaap, and Henry Houlden. All authors reviewed and commented on the final draft of the manuscript.

## Supporting information


**SUPP. TABLE S1**. Detailed genotype and phenotype of the newly reported patients.

Supporting information.

## Data Availability

All data generated or analyzed during this study can be found in the online version of the article on the publisher's website. All novel variants reported have been deposited in LOVD at https://databases.lovd.nl/shared/variants/ITPA.
