# Supplementary material for: Clinico‐radiological features, molecular spectrum, and identification of prognostic factors in developmental and epileptic encephalopathy due to inosine triphosphate pyrophosphatase (ITPase) deficiency
Source: Hum Mutat. 2022 Jan 12;43(3):403–19. doi: 10.1002/humu.24326 (PMC9152572; doi:10.1002/humu.24326)
Supplement: Supplementary file 2 — Supporting information. [file HUMU-43-403-s002.pdf]

Supplementary Material

1. Supplementary Methods

2. Supplementary Results

3. Supplementary Figures

4. Supplementary Tables

5. Supplementary References

1. Supplementary Methods

Subjects enrolment

The patients investigated in this study were enrolled at several different international research centers and hospitals: Al-Jawhara Centre for Molecular Medicine, Genetics and Inherited Disorders - Arabian Gulf University, Bahrein; Boston Children's Hospital, USA; Cantonal Hospital Lucerne, Switzerland; Children's Hospital of Eastern Ontario, Canada; Children's Memorial Health Institute of Warsaw, Poland; Duke University Hospital, Durham, USA; Dr. Sami Ulus Training and Research Hospital for Maternity and Children, Turkey; Hospital Clínic – IDIBAPS – CIBERER of Barcelona, Spain; Hospital Sant Joan de Déu, Spain; Howard Hughes Medical Institute – University of California, USA; Mashhad University of Medical Sciences, Iran; Mayo Clinic, USA; National Research Centre of Cairo, Egypt; Adult and Paediatric New Zealand National Metabolic Service, Starship Children's Hospital, New Zealand; Nicklaus Childrens Hospital, USA; King Faisal Specialist Hospital, Saudi Arabia; Tulane University School of Medicine, USA; UCL Queen Square Institute of Neurology, UK; VU University Medical Center Amsterdam, Netherlands; Zentrum für Kinder und Jugendmedizin Heidelberg - Universitätsklinikum Heidelberg, Germany. Informed written consent for genetic testing and publication of the clinical information, including clinical pictures, was obtained from the parents or the legal guardians of all the enrolled subjects according to the Declaration of Helsinki. Participant samples and data were obtained from multiple Institutions and the study was locally approved by the relative Ethics Committees (Mayo Clinic, Rochester, 16-004682; King Faisal Specialist Hospital and Research Centre, (RAC: 2121053, 2120022, and 2161245); Technische Universitaet Muenchen (TUM), 5360/12 S; University College London (UCL), project ID: 07/N018, REC Ref: 07/Q0512/26). No IRB approval was necessary for retrospective data analysis of a single patient for the following Institutions: Alberta Children's Hospital, Calgary, Canada; Al-Jawhara Centre for Molecular Medicine, Kingdom of Bahrain; Center for Neurogenomics and Cognitive Research, VU University, The Netherlands; Children's Hospital, Cantonal Hospital Lucerne, Switzerland; Dr. Sami Ulus Training and Research Hospital for Maternity and Children; Emma Children's Hospital, Amsterdam Leukodystrophy Center, The Netherlands; The Children's Memorial Health Institute, Poland.

Genetic testing and data analysis

After standard DNA extraction, Next Generation Sequencing (NGS) panel for epileptic encephalopathies (P5 and P9) and trio or proband exome sequencing (ES) (P1-4, P6-8, P10-28) were performed. NGS panels and ES were carried out as previously described (Ciara et al., 2018; Dunn et al., 2020; Guillen Sacoto et al., 2020; Kremer et al., 2016; Tarailo-Graovac et al., 2016). QC statistics with FastQC (http://www.bioinformatics.bbsrc.ac.uk/projects/fastqc) was used to assess the quality of the sequence reads. Reads alignment to the reference human genome (hg19, UCSC assembly, February 2009) was performed through BWA with default parameters (Li and Durbin, 2009). HaplotypeCaller algorithm within the GATK package was used for the recalibration of the quality score and for indel realignment and variant calling (DePristo et al., 2011; McKenna et al., 2010). Variants were then annotated with ANNOVAR (Wang et al., 2010). After being filtered out for minor allele frequency (MAF) ≤ 0.001 in genomic databases (in-house database of 16,500 exomes, GnomAD, GME, Iranome, and Ensembl), the predicted impact of the candidate variants on protein structure and function was evaluated through several in silico tools. These included: Combined Annotation Dependent Depletion (CADD, https://cadd.gs.washington.edu), Mutation Taster (http://www.mutationtaster.org), Mutation Assessor (http://mutationassessor.org/r3/), Sorting Intolerant From Tolerant (SIFT, https://sift.bii.a-star.edu.sg), Polyphen-2 (http://genetics.bwh.harvard.edu/pph2/), and Human Splice Finder (http://umd.be/Redirect.html). The most interesting candidate variants were confirmed through Sanger sequencing, which was performed according to standard procedures (Tarailo-Graovac et al., 2016). Sanger sequencing was also performed for parental segregation analysis. chromosomal microarray analysis (CMA) was performed in P2, P3, and P27 as previously described (Redon et al., 2009) and the identified deletions were interpreted according to the DECIPHER database (https://decipher.sanger.ac.uk). All variants are reported according to RefSeq NM_033453.3, GenBank NC_000020.11.

Computational biochemistry

A model based upon the ligand-bound structure PDB:2J4E [https://www.sciencedirect.com/science/article/pii/S0021925818384035] was constructed by adding two loops from the higher resolution unbound structure PDB:2CAR

[http://dx.doi.org/10.1074/jbc.M609838200]. The model was energy minimised in PyRosetta using 18 FastRelax cycles constrained to the electron density of PDB:2J4E, the rdkit_to_params Python module for ligand parameterisation [https://advances.sciencemag.org/content/7/16/eabf8711], and ref2015 scorefunction [https://pubs.acs.org/doi/abs/10.1021/acs.jctc.7b00125]. For each missense, after the introduction of the mutation, a 12Å neighbourhood was energy minimised. The scripts and models are available at https://doi.org/10.5281/zenodo.4818435.

2. Supplementary Results

Genetic details of the 1.1 Mb deletion in Family 2

In addition to ITPA, this maternal deletion (hg19, chr20: 2,816,108 – 3,955,033; https://www.deciphergenomics.org/search/genes?q=20%3A2816108-3955033) includes *AVP* (OMIM *192340), *DDRGK1* (OMIM *616177), *PANK2* (OMIM *606157), and *SLC4A11* (OMIM *610206). Heterozygous variants in *AVP* and *SLC4A11* cause autosomal dominant neurohypophyseal diabetes insipidus (OMIM #125700) and Corneal dystrophy, Fuchs endothelial, type 4 (OMIM #613268), respectively. Biallelic variants in the remaining genes are associated with autosomal recessive conditions: *DDRGK1* (Spondyloepimetaphyseal dysplasia, Shohat type - OMIM #602557); *PANK2* (HARP syndrome – OMIM #607236; Neurodegeneration with brain iron accumulation 1 – OMIM #234200); *SLC4A11* (Corneal endothelial dystrophy and perceptive deafness – OMIM #217400; Corneal endothelial dystrophy, autosomal recessive – OMIM #217700). Of note, no clinical features suggestive of corneal dystrophy or diabetes insipidus were observed in the patients (P2 and P3) in whom the deletion was detected.

Full deleted sequences of specific ITPA variants

c.264-607_295+1267del, p.(?)

g.3195320delGGTTCACGCCATTCTCCTGTCTCAGCCTCCCGAGTAGCTGGGACTACAGGCGCCCA
CCACCACACCTGGCTAATTTTTTGTATTTTTAGTAGAGACGGGGTTTCACCATGTTAGCCAGGAT
CGTCTCCATCTCCTGACCTCGTGATCTGCCCTTCTTGGCCTCCCAAAGTGCTGGGATTAGAGGCA
TGAGCCACCGCGCCCGGCCTGTATTTATTTATTTTTGGTGTGTGAGACAGGGTCTCAGTCTGTCG
CTGAGGTAGGAGTGCAGTGGTGTGATCACAGCTCACTCCCTGGGCTCAAGCTATCCACCTGCCT
CAGCCTCCCAAGTAGCTGGGACTGCAGGCACATGCCTAGCCAGTTTTTTTGCATTTTTTGTAGA
GATAGGATTTTGCTACGTTGTCCAAGCTTTTCTCAAACTCCTGAGCTCAAGCGATCCGCCTGCCT
TAGCCTCCCAAAGTGCTGGGATTATAGGCGGGACCCACCTCGCCTGGCTCTTGGAGGCATTCTT
TTTCATTTCCCCTTGGCTGTCAATAGGGAACAGCCTGGAAAAGGTGGTAAGAAGATCCAGCTGC
TCCTGGTGTCTGACTGTCCTTTCTTTCTCTTGCAGAAAGTGGTTTCTGGAGAAGTTAAAGCCTGA

AGGTATTATCTGCTTTGTTTCTTCCCTGGATCTGTTGGGAATGAGAATCTGGGCTTTGTCTAGGG

GAGGGACCCCAACTAGTAATCAGGAGTCCCAGGTTCTAGCCCCCACCATGGAGCCTCCACCTC

ATTTTCCCCATCTAGCACCTGCAGCTCGTACCCTGTACTCCAGAGAGCACTGTGTGGGGGAGGG

ATGATGGGAGCTGCAGGGTTGAGGAACAAAGGGGCTGTACTTAATTCTGGGTTTCAAGAGGAA

CTTGGCCCTTGTGACTGCCTTAGCCAGGGCCTGCCCTGGGAGTTGGAGGACTGCCCCTCCTGGA

CCCCAGCAGTGCATATCCTGTCACCCACAGTTGCAAGCCAAAATGTCCCCTCTTTATGTGCTTTT

ATTTATTTAGAGACAGGGTCTCACTCTGTTGCCCAGGCTGGAGTACACCGGTGTGATCTCGACT

CACTGCAACCTCTGCCTCTTGGGTTCAAGCGATTCTCCTGCCTTAGCCTTCCAAGTAGCTGGGAT

TACAGGCGCCCACCACCACGCCCTGCTAATTTTTGTATTTTTAGTAGAGAGGGGGTTTCACCAT

GTTGGCCAGGCCAGTCTCGAACACCTGACCTCAAGTGATCCGCCTACCTTGGCCTCCCAGAGTG

CTGGGATTACAGATGTGAGTCACAACACCCAGCCTATTTGTTTACTTATTTATATTTTTGAG

c.456_488+7del, p.(?):

g.3202528delGGACCCCTGCTTTCAGCCTGATGGATATGAGCAGACGTAA.

3. Supplementary Figure

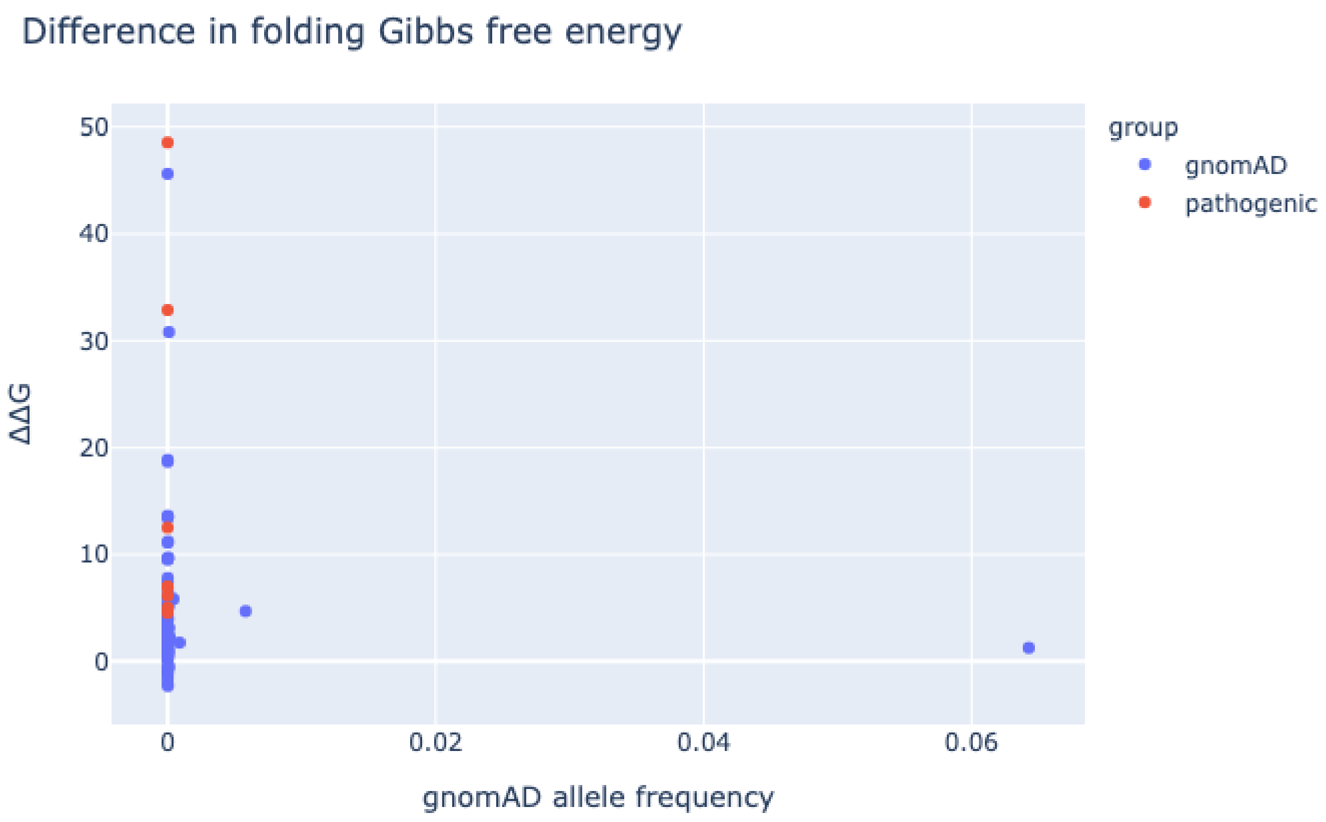

SUPP. FIGURE S1. Impact of *ITPA* missense variants on protein folding. The graph illustrates the difference in folding Gibbs free energy for pathogenic ITPA missense variants in comparison to variants present in healthy individuals (gnomAD, https://gnomad.broadinstitute.org/).

4. Supplementary Tables

SUPP. TABLE S2. MRI characteristics, Early MRI scans (≤ 4 months).

**Patient / Family**	**P2 / F2**	**P2 / F2**	**P3 / F2**	**P12 / F11**	**P24 / F20**	**P26 / F20**	**P27 / F21**
Age at MRI	2.5 months	4 months	6 days	3 weeks	3 months	1.5 months	3 months
Myelination	normal	normal	normal	normal	normal	normal	slightly delayed
Cerebral cortex	-	-	-	-	-	-	-
Basal nuclei/thalami	-	-	-	-	-	-	-
Cerebral hemispheric WM	-	-	-	-	-	-	-
Corpus callosum	normal for age	normal for age	normal for age	normal for age	normal for age	normal for age	normal for age
ALIC	-	-	-	-	-	-	-
PLIC	-	-	-	no low signal	+	-	+
Midbrain	-	-	-	-	-	-	-
Pons	-	+, CTT	-	-	-	-	-
Medulla	-	-	-	-	-	-	-
SCP	-	-	-	-	-	-	-
MCP	-	-	-	-	-	-	-
Cerebellar cortex	-	-	-	-	-	-	-
Cerebellar WM	-	-	-	-	+	-	-
Hilus dentate n.	-	-	-	-	-	-	-
Cerebral atrophy	slight	mild	no	no	slight	no	slight
Cerebellar atrophy	no	no	no	no	no	no	slight
Diffusion restriction	no	globus pallidus, PLIC, brachium inf. colliculus, CTT in midbrain, pons and medulla, decussation SCP, SCP, hilus dentate n.	no	globus pallidus, PLIC, left crus cerebri, decussation SCP, SCP, hilus dentate n., cerebellar WM, MCP, ICP	not done	Not available	OR, PLIC, crus cerebri
Contrast abnormalities	not administered	not administered	not administered	not administered	not administered	not administered	not administered
MR spectroscopy	not done	no lactate elevation	no lactate elevation	not done	not done	not done	no lactate elevation
Extra features				dilated inferior horns, rarefaction of temporal poles			

+ indicates abnormal T2 signal; **-** indicates no T2 signal abnormalities; OR, optic radiation; ALIC, anterior limb of the internal capsule; CTT, central tegmental tracts; ICP, inferior cerebellar peduncle; MCP, middle cerebellar peduncle; n., nucleus; PLIC, posterior limb of the internal capsule; SCP, superior cerebellar peduncle; WM, white matter

SUPP. TABLE S3. MRI characteristics, Intermediate MRI scans (4 - ≤ 8 months).

**Patient / Family**	**P1 / F1**	**P5 / F4**	**P6 / F5**	**P8 / F7**	**P9 / F8**	**P14 / F13**	**P28 / F23**
Age at MRI	6 months	4.5 months	4.5 months	6 months	4.5 months	5 months	8 months
Myelination	moderately delayed	normal	slightly delayed	mildly delayed	mildly delayed	slightly delayed	moderately delayed
Cerebral cortex	-	-	-	-	-	-	-
Basal nuclei/thalami	-	-	+, globus pallidus	-	-	-	-
Cerebral hemispheric WM	-	-	-	-	-	-	-
Corpus callosum	normal	normal	normal	thin	thin	thin	thin
ALIC	-	-	+	-	-	-	-
PLIC	+	+	+	+	+	+	-
Midbrain	+, decussation SCP, left crus cerebri	-	-	-	-	-	-
Pons	-	-	-	-	-	-	-
Medulla	-	-	-	-	-	-	-
SCP	-	-	-	-	-	-	-
MCP	+	-	-	-	-	+	-
Cerebellar cortex	-	-	-	-	-	-	-
Cerebellar WM	-	-	-	-	-	+	-
Hilus dentate n.	+	-	-	-	-	-	-
Cerebral atrophy	no	no	mild	mild	moderate	mild	moderate
Cerebellar atrophy	no	no	no	no	no	no	no
Diffusion restriction	OR, PLIC, decussation SCP	not done	globus pallidus, PLIC, crus cerebri, decussation SCP, SCP, hilus dentate n., pyramids	OR, globus pallidus, PLIC, crus cerebri, CTT in pons, decussation SCP, SCP, hilus dentate n., ICP	OR, globus pallidus, PLIC, crus cerebri, brachium inf. colliculus, CTT in pons, decussation SCP, SCP, hilus dentate n., cerebellar WM, ICP	OR, globus pallidus, ALIC, PLIC, crus cerebri, brachium inf. colliculus, decussation SCP, SCP, hilus dentate n., cerebellar WM	not done
Contrast abnormalities	no	not administered	not administered	not administered	no	no	not administered
MR spectroscopy	not done	not done	lactate elevation	not done	not done	not available	not done
Extra features	PLIC and left cerebral peduncle rarefied		several Virchow Robin-spaces		several Virchow Robin-spaces		

+ indicates abnormal T2 signal; **-** indicates no T2 signal abnormalities; ALIC, anterior limb of the internal capsule; CTT, central tegmental tracts; ICP, inferior cerebellar peduncle; MCP, middle cerebellar peduncle; n., nucleus; OR, optic radiation; PLIC, posterior limb of the internal capsule; SCP, superior cerebellar peduncle; WM, white matter.

SUPP. TABLE S4. MRI characteristics, Late MRI scans (> 8 months).

Patient / Family	P1 / F1	P1 / F1	P1 / F1	P2 / F2	P4 / F3	P10 / F9
Age at MRI	1 year	1.7 years	2.8 years	1.7 years	1.4 years	4.5 years
Myelination	moderately delayed	moderately delayed	moderately delayed	severely delayed	severely delayed	moderately delayed
Cerebral cortex	-	-	-	-	-	-
Basal nuclei/thalami	+, globus pallidus	-	-, thalamus atrophic	-, thalamus atrophic	-	-
Cerebral hemispheric WM	-	-	-	-	-	-
Corpus callosum	thin	thin	thin	thin	thin	thin
ALIC	-	-	-	-	-	-
PLIC	+	-	-	+	-	+
Midbrain	-	-	-	+, WM around red nucleus	-	-
Pons	-	-	-	+, CTT	-	-
Medulla	-	-	-	+, everything except inferior olivary n.	-	-
SCP	-	-	-	-	-	-
MCP	+	-	-	-	-	-
Cerebellar cortex	-	-	-	-	-	-
Cerebellar WM	-	-	-	+, peridentate WM	-	-
Hilus dentate n.	-	-	-	-	-	-
Cerebral atrophy	mild	moderate	moderate-severe	severe	moderate-severe	moderate-severe
Cerebellar atrophy	slight	mild	mild	moderate	no	no
Diffusion restriction	pericentral and parietal WM, OR, ALIC, PLIC, decussation SCP, MCP	no	no	cerebral hemispheric WM, corpus callosum, ALIC, PLIC, crus cerebri, WM around red nucleus, brachium inf. colliculus, CTT in pons, decussation SCP, SCP, cerebellar WM, ICP	OR, ALIC, PLIC, CTT in pons, ICP	not done
Contrast abnormalities	not administered	no	not administered	not administered	not administered	not administered
MR spectroscopy	not done	no lactate elevation	not done	not done	no lactate elevation	not done
Extra features				tram-track T2-hyperintensity PLIC	rarefaction of temporal poles	

+ indicates abnormal T2 signal; **-** indicates no T2 signal abnormalities; ALIC, anterior limb of the internal capsule; CTT, central tegmental tracts; ICP, inferior cerebellar peduncle; MCP, middle cerebellar peduncle; n., nucleus; OR, optic radiation; PLIC, posterior limb of the internal capsule; SCP, superior cerebellar peduncle; WM, white matter.

5. Supplementary References

Ciara, E., Rokicki, D., Lazniewski, M., Mierzewska, H., Jurkiewicz, E., Bekiesińska-Figatowska, M., Piekutowska-Abramczuk, D., Iwanicka-Pronicka, K., Szymańska, E., Stawiński, P., Kosińska, J., Pollak, A., Pronicki, M., Plewczyński, D., Płoski, R., Pronicka, E. (2018) Clinical and molecular characteristics of newly reported mitochondrial disease entity caused by biallelic PARS2 mutations. *J Hum Genet, 63,* 473-485. https://doi.org/10.1038/s10038-017-0401-z

DePristo, M.A., Banks, E., Poplin, R., Garimella, K.V., Maguire, J.R., Hartl, C., Philippakis, A.A., del Angel, G., Rivas, M.A., Hanna, M., McKenna, A., Fennell, T.J., Kernytsky, A.M., Sivachenko, A.Y., Cibulskis, K., Gabriel, S.B., Altshuler, D., Daly, M.J. (2011) A framework for variation discovery and genotyping using next-generation DNA sequencing data. *Nat Genet, 43,* 491-498. https://doi.org/10.1038/ng.806

Dunn, P., Albury, C.L., Maksemous, N., Benton, M.C., Sutherland, H.G., Smith, R.A., Haupt, L.M., Griffiths, L.R. (2018) Next Generation Sequencing Methods for Diagnosis of Epilepsy Syndromes. *Front Genet, 9,* 20. https://doi.org/10.3389/fgene.2018.00020

Guillen Sacoto, M.J., Tchasovnikarova, I.A., Torti, E., Forster, C., Andrew, E.H., Anselm, I., Baranano, K.W., Briere, L.C., Cohen, J.S., Craigen, W.J., Cytrynbaum, C., Ekhilevitch, N., Elrick, M.J., Fatemi, A., Fraser, J.L., Gallagher, R.C., Guerin, A., Haynes, D., High, F.A., … Juusola, J. (2020) De Novo Variants in the ATPase Module of MORC2 Cause a Neurodevelopmental Disorder with Growth Retardation and Variable Craniofacial Dysmorphism. *Am J Hum Genet, 107,* 352-363. https://doi.org/10.1016/j.ajhg.2020.06.013

Kothur, K., Holman, K., Farnsworth, E., Ho, G., Lorentzos, M., Troedson, C., Gupta, S., Webster, R., Procopis, P.G., Menezes, M.P., Antony, J., Ardern-Holmes, S., Dale, R.C., Christodoulou, J., Gill, D., Bennetts, B. (2018) Diagnostic yield of targeted massively parallel sequencing in children with epileptic encephalopathy. *Seizure, 59,* 132-140. https://doi.org/10.1016/j.seizure.2018.05.005

Kremer, L.S., Danhauser, K., Herebian, D., Petkovic Ramadža, D., Piekutowska-Abramczuk, D., Seibt, A., Müller-Felber, W., Haack, T.B., Płoski, R., Lohmeier, K., Schneider, D., Klee, D., Rokicki, D., Mayatepek,

E., Strom, T.M., Meitinger, T., Klopstock, T., Pronicka, E., Mayr, J.A., Baric, I., Distelmaier, F., Prokisch, H. (2016) NAXE Mutations Disrupt the Cellular NAD(P)HX Repair System and Cause a Lethal Neurometabolic Disorder of Early Childhood. *Am J Hum Genet, 99,* 894-902. https://doi.org/10.1016/j.ajhg.2016.07.018

Li, H., & Durbin, R. (2009) Fast and accurate short read alignment with Burrows-Wheeler transform. *Bioinformatics, 25,* 1754-1760. https://doi.org/10.1093/bioinformatics/btp324

McKenna, A., Hanna, M., Banks, E., Sivachenko, A., Cibulskis, K., Kernytsky, A., Garimella, K., Altshuler, D., Gabriel, S., Daly, M., DePristo, M.A. (2010) The Genome Analysis Toolkit: a MapReduce framework for analyzing next-generation DNA sequencing data. *Genome Res, 20,* 1297-1303. https://doi.org/10.1101/gr.107524.110

Redon, R., & Carter, N.P. Comparative genomic hybridization: microarray design and data interpretation. (2009) *Methods Mol Biol, 529,* 37-49. https://doi.org/10.1007/978-1-59745-538-1_3

Tarailo-Graovac, M., Shyr, C., Ross, C.J., Horvath, G.A., Salvarinova, R., Ye, X.C., Zhang, L.H., Bhavsar, A.P., Lee, J.J., Drögemöller, B.I., Abdelsayed, M., Alfadhel, M., Armstrong, L., Baumgartner, M.R., Burda, P., Connolly, M.B., Cameron, J., Demos, M., Dewan, T., … van Karnebeek, C.D. (2016) Exome Sequencing and the Management of Neurometabolic Disorders. *N Engl J Med, 374,* 2246-2255. https://doi.org/10.1056/NEJMoa1515792

Wang, K., Li, M., Hakonarson, H. (2010) ANNOVAR: functional annotation of genetic variants from high-throughput sequencing data. *Nucleic Acids Res, 38,* e164. https://doi.org/10.1093/nar/gkq603
